# Outpatient Psychotherapy Reduces Health-Care Costs: A Study of 22,294 Insurants over 5 Years

**DOI:** 10.3389/fpsyt.2016.00098

**Published:** 2016-06-13

**Authors:** Uwe Altmann, Anna Zimmermann, Helmut A. Kirchmann, Dietmar Kramer, Andrea Fembacher, Ellen Bruckmayer, Irmgard Pfaffinger, Fritz von Heymann, Emma Auch, Rolf Steyer, Bernhard M. Strauss

**Affiliations:** ^1^Institute of Psychosocial Medicine and Psychotherapy, University Hospital Jena, Jena, Germany; ^2^Bavarian Association of Statutory Health Insurance Physicians, Munich, Germany; ^3^Psychotherapist in Private Practice, Feldafing, Germany; ^4^Medical Specialist for Psychosomatic Medicine and Psychotherapy in Private Practice, Munich, Germany; ^5^Qualitas GmbH, Munich, Germany; ^6^Institute of Psychology, Friedrich-Schiller-University Jena, Jena, Germany

**Keywords:** health-care utilization, mental disorder, outpatient psychotherapy, cost reduction, economic benefits

## Abstract

**Background:**

The project “Quality Assurance in Ambulatory Psychotherapy in Bavaria” (QS-PSY-BAY) focuses on the quality assurance of outpatient psychotherapy (OPT) in Germany in terms of symptom reduction and cost reduction under naturalistic conditions. In this study, we examined the effectiveness of psychotherapy in terms of pre–post cost reduction.

**Method:**

The health-care costs of *N* = 22,294 insurants over a 5-year period were examined in a naturalistic longitudinal design. Six participating health insurance funds provided data on costs related to inpatient treatment, outpatient treatment, drugs, and hospitalization and work disability days.

**Results:**

We found that the average annual total costs for inpatient and outpatient treatments as well as drug costs and work disability days increased from the second to the first year before OPT. Besides a large and significant reduction of work disability days (41.8%), hospitalization days (27.4%), and inpatient costs (21.5%) from the first year before versus the first year following OPT, we found evidence for long-term effects: the number of work disability days in the second year after OPT was lower (23.8%), and drug costs were higher than in the second year before OPT (41.5%).

**Conclusion:**

We conclude that OPT as a part of the health insurance system is an investment which can pay off in the future especially in terms of lower inpatient costs and work disability.

## Introduction

Health services research focuses on medical and psychotherapeutic treatments under naturalistic conditions. Because health expenditures are very large (e.g., in Germany, 11.3% of gross domestic product; see OECD Health Statistics 2014) and a considerable proportion of people suffer from a mental disorder [e.g., 38.2% in the EU; Ref. (1)], the examination of the monetary aspects of psychological treatments becomes increasingly relevant. Multiple reviews and meta-analyses (2–8) suggest that costs and medical treatment utilization after psychotherapy are reduced, and that – compared with medical treatment – psychotherapy is more effective in terms of symptom and cost reduction. In general, psychotherapy seems to be cost-effective, although the available reviews and meta-analyses usually refer to studies of health-care systems of different countries, various psychological approaches, and/or evaluations of different cost variables.

In this study, we focused on outpatient psychotherapy (OPT) in Germany. In general, little is known about the cost-effectiveness of OPT (3). The examination of the cost-effectiveness of psychotherapy is subject to the problem that studies come from various health services systems with different general conditions for OPT. The number of therapy sessions, e.g., provides one marked difference: in the USA, e.g., the median number of OPT sessions is 5, whereas in German, short-term psychotherapy comprises 25 sessions (9). For this reason, results related to therapy outcome or its cost-effectiveness are not directly comparable between countries.

In Germany, three approaches of psychological treatment are part of the health insurance system: cognitive behavioral psychotherapy (CBT), psychodynamic psychotherapy (PDP), and psychoanalytic (long-term) psychotherapy (PP). In all three approaches, the first five sessions are seen as “probatory” sessions in which patients’ psychological and social problems are clarified and the corresponding mental disorders are diagnosed. The subsequent therapy sessions must be requested from the health insurance fund. Usually, it is distinguished between short-term (up to 25 sessions) and long-term psychotherapies (50 sessions and more). Both forms can be extended in case of a complex mental disorder and therapy progress [for more details, see Ref. (10)].

Two issues will mainly be focused in health-care utilization research: the first is the examination of treatment costs related to specific mental disorders. For depressive patients, for example, Salize et al. (11) reported that the mean annual costs of medical care were €3849. For patients with alcoholic addiction, Stamm et al. (12) reported that the mean expenditure by a health insurance company on insurants with a one-time diagnosis in the reference year was €2888.97, whereas it was €5261.82 for chronic alcoholics. In their review of the costs of schizophrenia in Germany, Konnopka et al. (13) found that the direct annual costs per person varied between €14,000 and €18,000 and costs related to the loss of productivity varied between €25,000 and €30,000.

A second issue is the examination of cost reduction as a consequence of psychotherapy. Comparing the first year before with the first year after OPT, Kraft et al. (14) reported a 6.7% reduction of medical costs from €3717.92 to €3468.47. In addition, days of hospitalization were reduced between 1.8 and 19.8 (14–17). The number of work disability days as an aspect of indirect costs has also been studied. Multiple studies (15, 17–20) reported a decrease of 3.0–19.6 days, when comparing the first year before with the first year after OPT (for more details, see Table S1 in Supplementary Material).

Taken together, the studies mentioned above reported reductions in multiple cost factors in the context of OPT in Germany. The most recent study dates back to 2006, and all the sample sizes were under 700 insurants (see Table S1 in Supplementary Material). In these studies, missing data were not imputed, so that – due to list-wise deletion – selection bias can be assumed. Furthermore, the reduction of costs varies widely among studies, which could be due to small samples and distortions caused by high cost cases and/or sampling bias. An examination of a large and representative sample is needed to evaluate the quality of outpatient therapy in Germany in terms of cost reduction.

The project “Qualitätssicherung in der ambulanten Psychotherapie in Bayern” (“Quality Assurance in Ambulatory Psychotherapy in Bavaria;” QS-PSY-BAY) focuses on the quality assurance of German OPT under naturalistic conditions (21–23). We were interested in both, the effectiveness of OPT and monetary aspects like cost reduction. Previous analyses have focused on a subsample with *N* ≈ 1700 patients, including questionnaire data. The results lead to the following conclusions (21–26): under naturalistic conditions, OPT is effective in reducing symptom intensity and improving quality of life – even in the case of premature discontinuation; the dosage of psychotherapy obviously is adapted to treatment conditions and the severity of the patient’s illness.

In this study, we focus on the monetary aspect of OPT. Analyzing health insurance data of 22,294 persons with statutory health insurance, we examined inpatient costs, outpatient costs, drug costs, number of work disability days, and number of hospitalization days under naturalistic conditions. According to Kraft et al. (27), we hypothesized (1) that health-care costs increase in the time before a person will be treated with OPT and (2) that health-care costs should be reduced from pre- to posttreatment.

## Materials and Methods

### Sample

The sampling was performed by the *Kassenärztliche Vereinigung Bayerns* (KVB; Bavarian Association of Compulsory Health Insurance Physicians). A random sample of *N* = 38,338 persons was generated. Inclusion criteria were (1) the person was insured by one of the six participating statutory health insurance, (2) age over 18 years, (3) diagnosis of mental disorder (ICD10: F2–F6) except dementia (ICD10: F0) and addiction (ICD10: F1), and (4) treated with individual OPT. All inclusion criteria should apply for the reference quarter (billing date at third quarter of the year 2008). In the German health system at the time, the study was planned, addiction and dementia are not treated in an OPT setting. We selected 22,294 out of the 38,338 insurants whose start and end of psychotherapy could be identified based on cost data (see Data Preprocessing). Table 1 shows the distribution of gender, age, place of residence, and the frequency of the different psychotherapeutic approaches.

**Table 1:** **Distribution of age, gender, place of residence, and psychotherapeutic approach in the random sample with psychotherapy and in the examined subsample of patients, whose begin and end of therapy could be identified using cost data**.

	Random sample with OPT	Examined sample with OPT
Sample size	38,336	22,294
Men (%)	22.8	23.3
Women (%)	77.2	76.7
Age 18 to <35 (%)	24.7	24.3
Age 35 to <50 (%)	42.9	41.0
Age 50 to <65(%)	27.2	28.5
Age 65–110 (%)	5.2	6.2
Urban agglomeration (%)	48.2	46.0
Urbanized area (%)	25.3	26.2
Rural area (%)	26.6	27.8
Cognitive behavioral therapy (individual therapy) (%)	39.8	46.1
Psychodynamic psychotherapy (individual therapy) (%)	47.1	48.6
Psychoanalysis (individual therapy) (%)	13.1	5.3

It should be noted that the examined sample is rather heterogeneous regarding mental disorders and psychotherapeutic treatments. The distribution of psychotherapeutic approaches was CBT 46.1%, PDP 48.6%, and PP 5.3%. The 35.5% insurants additionally received various psychotropic drugs [antipsychotics (ATC Code: N05A), anxiolytics (N05B), hypnotics and sedaticies (N05C), antidepressants (N06A)]. The mean duration of psychotherapy was *M* = 31.3 sessions (Median = 25, *SD* = 31.3) lasting – on an average – for seven quarters (Median = 5, *SD* = 2.9), i.e., ~21 months. Depending on the psychological approach, the mean number of sessions was CBT, *M* = 30.8; PDP, *M* = 33.2; and PP, *M* = 94.7.

### Outcome Respectively Cost Variables

**Figure 1:**
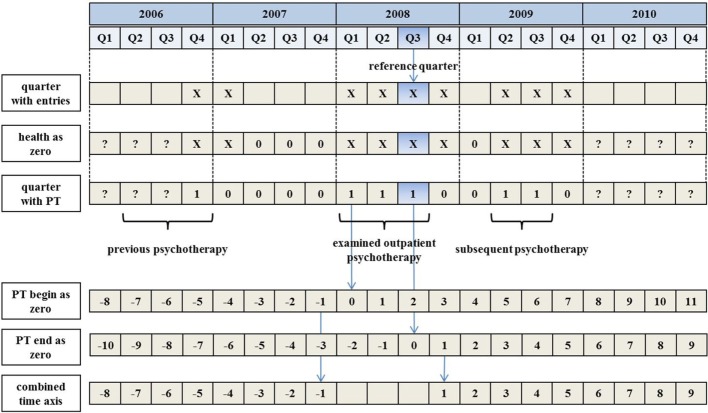
**Data preprocessing and computation of used time axis**.

The six participating health insurance funds provided inpatient treatment costs, drug costs, and information about hospitalization and work disability days. The KVB, on the other hand, provided outpatient treatment cost data for the sample. These data were matched by anonymous one-to-one identification number. We obtained information about demographic variables (e.g., gender, year of birth, and category for the domicile), inpatient and outpatient treatments (e.g., diagnosis, treatment, physician group of the treating physician/psychotherapist, treatment time point, and costs), prescription of drugs (e.g., drug type, price), and work disability days for the time interval from 2006 Q1 to 2010 Q3 (cf., Figure 1). We obtained no data about insurance status (e.g., date of change the health-care fund), family background, rehabilitation, and dental treatments.

### Data Preprocessing

First, for each cost variable, we aggregated the individual cost entries for each quarter. To identify which cost entry related to the quarter of interest, we used the performance date (not the payment date). If the time interval of work disability or hospitalization extended more than one quarter, we splitted the interval accordingly. With the quarterly aggregation, the background for a cost entry was lost. For example, a quarterly total of outpatient treatment costs could have been caused by flu, knee injury, as well as mental disorder treatment, or even by a mixture of treatments.

In a next step, we distinguished between healthy individuals and those whose health-care costs were not documented, for example, due to a change in their health-care fund or caused by death. In both cases, we did not find any entry for such persons in the quarter of interest. Hence, for each person, we identified the earliest entry across all variables (e.g., work disability days in 2006 Q4) and the latest entry (e.g., outpatient costs in 2009 Q4). Within this individual “trust interval” (in our example between 2006 Q4 and 2009 Q4), quarters without cost entries were set to 0 because we could assume that the person was healthy (see Figure 1). Empty quarters outside the trust interval were coded as missing value (see Figure 1).

We then identified the beginning and the end of OPT for each person (Figure 1), assuming that quarter *t* is the first quarter of the OPT if quarter *t* − 1 contained no entry for a treatment. Similarly, we assumed that the therapy ended in quarter *t* if quarter *t* + 1 revealed no entry for OPT. The time interval of the examined OPT had to include the reference quarter 2008 Q3; otherwise, the insurant was excluded from data analysis.

Furthermore, we aligned the individual time courses of the cost variables to the beginning and end of OPT. We generated a time axis, in which negative values indicated quarters before OPT and positive values quarters after OPT. Quarters comprising OPT were excluded in this time axis (see Figure 1, line “combined time axis”). Using such an aligned time course of health-care costs, we computed annual totals for the second year before OPT (quarter −8 to −5), the first year before OPT (quarter −4 to −1), the first year after OPT (quarter 1–4), and the second year after OPT (quarter 5–8).

### Missing Data

We had complete sociodemographic and cost data for the reference quarter 2008 Q3 for all insurants. The number of missing cost data increased with the temporal distance from the reference quarter (number of insurants with missing data: 11,351 of 22,294 in the eighth quarter before OPT, 2517 in fourth quarter before, and 0 in first quarter before and in first quarter after OPT, 4360 in fourth quarter after OPT, and 17,993 in eighth quarter after OPT). For parameter estimation, we used the full-information maximum likelihood (FIML) method using all available sociodemographic and cost variables as covariates, which is comparable with missing-at-random imputation.

### Data Analysis

#### Representativeness of Sample

As mentioned above, in the reference quarter (2008 Q3), random sample of 38,338 insurants was treated with OPT. From this sample, *N* = 22,294 insurants were selected for which the beginning and the end of therapy could be identified based on the available cost data. Using nominal regression, we examined systematic differences between the entire sample and the analyzed subsample. The potential predictors for selection effects were age group, gender, place of residence, and psychotherapeutic approach. The outcome variable was a binary variable, which indicates with 1 and 0 that a person was included in the examined sample. Based on χ^2^-statistics of nominal regression, we computed Cramer’s *V* as an effect size measure. According to Rea and Parker (28), Cramer’s *V* can be interpreted as negligible (0–0.1), weak (0.1–0.2), moderate (0.2–0.3), relatively strong (0.4–0.6), strong (0.6–0.8), or very strong (0.8–1.0). Furthermore, the fit of nominal regression in terms of Nagelkerke *R*^2^ was considered. According to Cohen (29), *R*^2^ can be interpreted as small (0.0196–0.13), moderate (0.13–0.26), and large (0.26–1.0).

#### Comparison of Annual Totals

To study the effect of psychotherapy in terms of cost reduction, we computed annual totals for each cost variable. We focused on the second and the first year before the beginning of OPT, as well as on the first and the second year after end of OPT. An individual annual total represents the sum of the corresponding quarterly totals. The averages of the annual totals were compared conducting a *t*-test for paired samples. The level of significance was α = 0.05.

## Results

### Representativeness of Sample

Using nominal regression and the predictors’ age, gender, place of residence, and psychotherapeutic approach, we examined the representativeness of our examined sample of *N* = 22,294 (for descriptive statistics, see Table 1). Except place of residence all variables significantly predicted the fact if a person is or is not part of the examined subsample (gender: $\chi_{\text{df}=1}^{2}=6.5,$ *p* = 0.011, Cramer’s *V* = 0.013; age: $\chi_{\text{df}=3}^{2}=105.8$, *p* < 0.001, Cramer’s *V* = 0.053; place of residence: $\chi_{\text{df}=2}^{2}=5.2$, *p* = 0.076, Cramer’s *V* = 0.012; and psychological approach: $\chi_{\text{df}=2}^{2}=2853.3$, *p* < 0.001, Cramer’s *V* = 0.273). Despite significant differences, the corresponding effect sizes for gender, age group, and place of residence can be interpreted as negligible (Cramer’s *V* < 0.1), whereas the psychotherapeutic approach seems to be the only relevant selection effect since the effect size can be interpreted as moderate. The model fit of nominal regression points into the same direction: Nagelkerke *R*^2^ was 0.107, indicating that 10.7% of the outcome variable’s variance can be explained by the regression with age group, gender, place of residence, and psychotherapeutic approach as predictors. Running the model without the treatment variable, Nagelkerke *R*^2^ was only *R*^2^ = 0.011, which is smaller than the cut-off value for small effects. It also means that the psychotherapeutic approach explains 9.6 = 10.7 − 1.1% of the variance and that the selection process is mostly driven by the psychotherapeutic approach. On average, psychoanalytic long-term treatment was underrepresented in our examined subsample (see Table 1).

### Costs before and after Outpatient Psychotherapy

**Figure 2:**
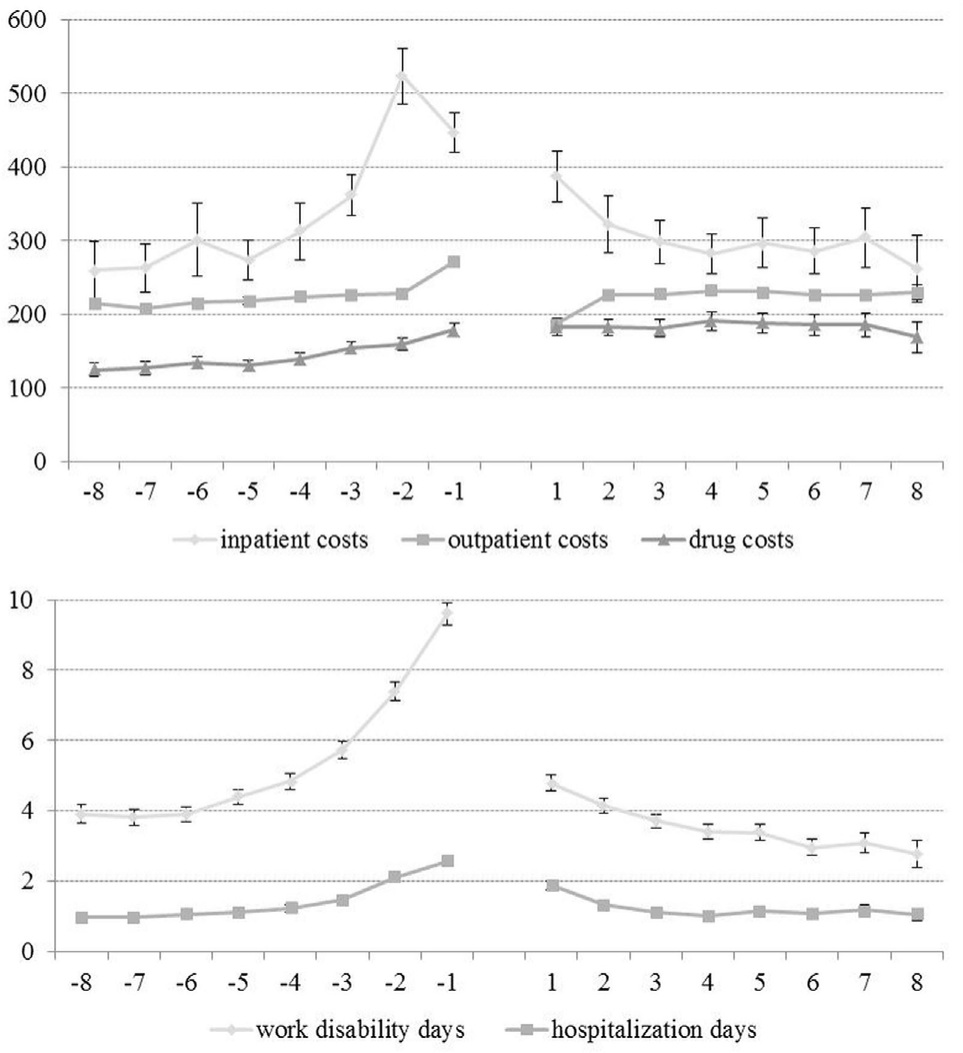
**Time course of cost variables (means of quarterly totals and 95% confidence intervals; negative time values indicate quarters before outpatient psychotherapy and positive time values quarter after psychotherapy; at each time point, *N* = 22,294)**.

**Figure 3:**
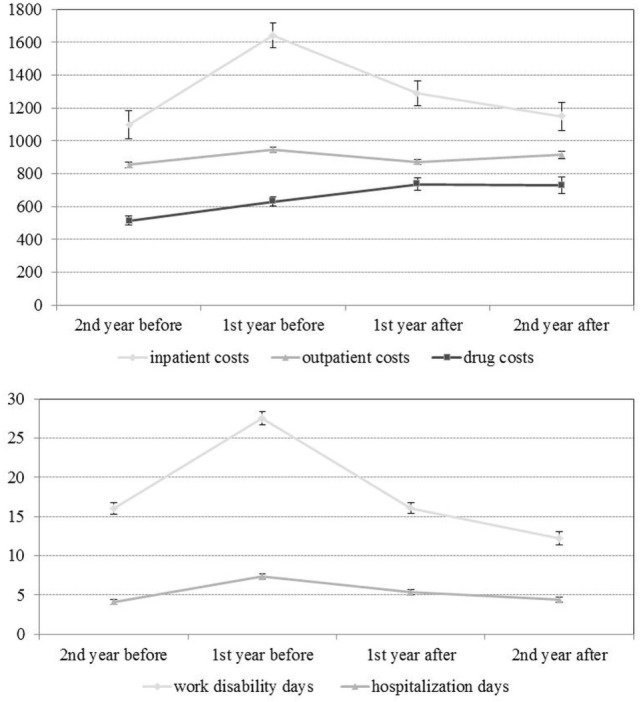
**Annual costs before and after outpatient psychotherapy (means and their 95% confidence intervals; at each time point, *N* = 22,294)**.

The means of the quarterly totals are shown in Figure 2, and the means of the annual totals in Figure 3. It can be seen that work disability days and inpatient cost increased considerably before OPT and decreased to their initial values following OPT.

In Table 2, the means of the annual totals are listed. Comparing 1 year before and 1 year after psychotherapy, the inpatient costs, outpatient costs, work disability days, and hospitalization days were reduced significantly (see also Table 3). Furthermore, in the second year after psychotherapy, the work disability days were significantly lower than in the second year before psychotherapy. In the second year after psychotherapy, days of hospitalization, inpatient costs, and outpatient cost were on similar level as in the second year before psychotherapy. In contrast to all the other variables, we found significant growth in drug costs. Taking inpatient, outpatient, and drugs costs together, the total cost reduced from the first year before to the first year after OPT, which means that the reduction of inpatient and outpatient costs predominated the growth of drug costs.

**Table 2:** **Averages of annual total costs before and after outpatient psychotherapy**.

Averages of annual totals	Second year before	First year before	First year after	Second year after
Inpatient costs (€)	1097.36	1642.90	1289.05	1147.39
Outpatient costs (€)	854.40	947.41	872.21	913.38
Drug costs (€)	513.87	630.04	735.28	727.17
Total costs (€)	2465.63	3220.35	2896.54	2787.95
Work disability days	16.01	27.57	16.04	12.20
Hospitalization days	4.08	7.35	5.33	4.42

*The sample size is *N* = 22,294*.

**Table 3:** **Comparison of annual totals before and after outpatient psychotherapy**.

	diff	(*SE*)	diff in %	*ES*
**Second before vs. first after**				
Inpatient costs (€)	545.54***	(50.94)	49.7	0.071
Outpatient costs (€)	93.01***	(8.08)	10.9	0.077
Drug costs (€)	116.17***	(12.97)	22.6	0.060
Total costs (€)	754.72***	(54.22)	30.6	0.093
Work disability days	11.56***	(0.44)	72.2	
Hospitalization days	3.27***	(0.19)	80.1	
**First before vs. first after**				
Inpatient costs (€)	−353.84***	(49.17)	−21.5	0.048
Outpatient costs (€)	−75.20***	(8.03)	−7.9	0.063
Drug costs (€)	105.23***	(17.34)	16.7	0.041
Total costs (€)	−323.80***	(53.79)	−10.1	0.040
Work disability days	−11.53***	(0.48)	−41.8	0.161
Hospitalization days	−2.02***	(0.19)	−27.4	0.071
**Second before vs. second after**				
Inpatient costs (€)	50.04^n.s.^	(59.55)	4.6	0.006
Outpatient costs (€)	58.98***	(12.86)	6.9	0.031
Drug costs (€)	213.30***	(25.84)	41.5	0.055
Total costs (€)	322.32***	(69.79)	13.1	0.031
Work disability days	−3.82***	(0.53)	−23.8	0.048
Hospitalization days	0.35^n.s.^	(0.21)	8.6	0.011

*The sample size is *N* = 22,294*.

***p < 0.001.

*^n.s.^*p* ≥ 0.05*. *diff, absolute difference; diff in %, difference as percent of pretreatment value; ES, effect size*.

## Discussion

Outpatient psychotherapy is effective in terms of symptom reduction and the improvement of quality of life (23). Little is known about the cost-effectiveness of OPT (3), especially related to recent and representative data. Accordingly, we examined the cost reduction in the context of OPT in Germany under naturalistic conditions.

### Increasing Heath-Care Cost before Outpatient Psychotherapy

Looking at the quarterly means before the OPT, we found that the outpatient costs are relatively stable, whereas inpatient costs, drug costs, hospitalization days, and work disability days began to increase around four quarters before OPT (Figure 2). This finding can be interpreted in different ways. First, it is possible that an insurant with an untreated (or undiagnosed) mental disorder might be “going around” and tries out various (inpatient and/or pharmacological) treatments until he/she finds the adequate outpatient psychological treatment. Another interpretation is that a mental disorder might be triggered by a hospitalization (30) and subsequently treated within an outpatient setting. Additionally, the time courses of cost variables indicate that an outpatient psychotherapeutic treatment might also be a continuation of inpatient psychotherapeutic treatments. We assume that all explanations might partly apply. However, future research should take time courses of cost variables into the account, depending on pretreatments before OPT (e.g., psychotherapy of PTSD after ICU stay).

Furthermore, we found that costs during the time to find an adequate treatment of mental disorder are high, for example, the annual inpatient cost increased from €1097.36 to €1642.90 (mean annual totals) and the annual work disability days from 16.01 to 27.57 days (see Table 2). These annual costs were smaller than the annual means reported by Kraft et al. (27), but similar related to the cost medians. The difference between both studies might be caused by a methodological issue: the small sample of Kraft et al.’s (27) study (*N* = 708) is more sensitive to high cost cases than the large sample (*N* = 22,294) in our study. However, the study by Kraft et al. (27) and our results suggest that an untreated mental disorder is associated with increasing costs. This finding affects the issue of waiting times for OPT, which is currently discussed in Germany [according to recent studies, e.g., Ref. (31), the average waiting time is 3 months from the first contact to the first session, with a considerable variance depending on the geographical region]. Furthermore, studies should focus on the dependency between increasing costs and the time between diagnosis of mental disorder and beginning of OPT. It can be assumed that health-care costs could be reduced by shorter waiting periods (2).

### Reduction of Health-Care Cost after Outpatient Psychotherapy

For the examination of cost reductions, we compared the average annual totals 1 year before and 1 year after OPT. Outpatient costs were reduced by €75.20 (7.9% of the annual total 1 year before psychotherapy), inpatient costs by €353.84 (21.5%), hospitalization days by 2.02 days (27.4%), and work disability days by 11.53 days (41.8%) (Table 3). These cost reductions differ from the findings of other studies, which could be explained by a different distribution of the psychological approaches and the sample composition. Breyer et al. (19) and Keller et al. (17), for example, reported less reduction in work disability days for patients treated with psychoanalysis (8.6 and 6.4 days, respectively). Jacobi (16), who studied only CBT for anxiety disorder, reported a higher number than our study (19.8 days). Kraft et al. (14) examined only CBT and PDP and reported less reduction of medical costs (€249.45, respectively, 6.7%). However, like other studies, our study shows that OPT is cost-effective in terms of cost reduction.

Contrary to these findings, drug costs increased significantly by €105.23 (respectively, 16.7%) between the first year before OPT and the first year after OPT (see Table 3) in our study. This suggests that some persons probably started with a pharmacological treatment after OPT. Possibly, this finding reflected various price increases. Future studies should also examine the frequency and conditions of such subsequent treatments (e.g., due to which disorder, severity of disorder, risk of relapse, therapeutic failure of OPT, etc.). However, the reduction of the inpatient and outpatient cost was larger than the increase of the drug cost, resulting in a total cost reduction of €32.80 (10.1%).

We also found evidence for long-term effects of OPT (Table 3): in the second year after OPT, the annual work disability days were lower than in the second year before therapy. This suggests that OPT not only reduces the costs but also increases psychosocial functioning of a patient with respect to lowering the number of work disability days.

Altogether, our results are in line with the review of Gabbard et al. (4), who stated that much of the impact of OPT is related to reductions in inpatient treatment and decreases in work impairment. Despite support for a cost reduction, one should not forget that monetary aspects are not the only criterion for the evaluation of a psychotherapeutic treatment. Effectiveness in terms of symptom reduction and improvement of life quality still should be the primary criterion; accordingly, it would be of interest to compare treatments with similar effectiveness related to their monetary aspects (2).

### Limitations of the Study

In this study, we examined cost data referring to a time interval of 5 years provided by multiple health insurance funds. Starting from a random sample (*N* = 38,338) of Bavarian insurants who were treated with OPT, we selected a sample (*N* = 22,294) where the beginning and the end of the OPT could be clearly identified. With regard to gender, age, and place of residence, this subsample did differ in a negligible way from the initial random sample. We found that gender and age group significantly predicted the inclusion into the examined sample, but the corresponding effect sizes were almost negligible (Cramer’s *V* < 0.1), whereas PP was underrepresented in our examined sample (Cramer’s *V* = 0.27, respectively, moderate effect size). This limitation might be caused by the fact that PP usually comprises much more therapy sessions than CBT and PDP. Accordingly, the beginning *and* the end of therapy might have been underrepresented within our 5-year study interval leading to an exclusion of more patients. This problem could only be solved within study intervals lasting longer than 5 years.

The identification of the beginning and the end of the therapies might be a subject of discussion, since the health insurance funds did not provide explicit information. The start and the end of therapy were identified by detecting the quarters in which no OPT was accounted for (Figure 1). This method fails if an insured person waits for a therapy extension for longer than one quarter. However, we assumed that such cases are very rare and that the resulting bias is tolerable given our large sample.

Another problem concerns the correct identification of the quarter in which a person is “healthy” in the sense of causing no costs for the insurance company. In our database, an insured person is only “visible” if he/she causes treatment costs. Before the earliest entry and after the last entry, it could be (1) that the insurant was “healthy” and causing no costs or (2) that he/she changed the health-care fund and prompted no further entries under the “old” identification number. This could be a general problem for health insurance data analysis, since the time-dependent sample sizes of Kraft et al. (14) suggested that they had the same problem. All reported problems are quite typical for the analysis of health insurance fund data as we realized throughout the project [cf., Ref. (23)].

Compared with other studies related to monetary aspects of OPT in Germany, our study has at least two strengths: first, our large sample (*N* = 22,294) is unique compared with other studies (*N* < 700; see Table S1 in Supplementary Material). The larger size of our sample contributes to the fact that our estimations will be more accurate, e.g., in terms of smaller standard errors. Second, our data came from more than one health insurance fund. This avoids selection bias, since sociodemographic characteristics and the prevalence of chronic diseases differ between health insurance funds (32). As a consequence, in a sample confined to one health insurance fund, the average costs might be biased.

One important aspect in the discussion relates to the question if and how treatment costs and conditions may change over time: during our study, six different legal initiatives relevant to health care were passed. Such changes cannot be avoided in naturalistic long-term sectional studies about health-care costs. In addition, inflation [in Germany, 1.5% per year between 2000 and 2010 (33)] and changes in the *per capita* health expenditure [in Germany, 3% growth per year between 2000 and 2010 (34)] can be seen as confounders. Some researchers adjust their data by applying cost reduction with regard to inflation and growth of *per capita* health expenditures. We decided to set such an adjustment aside to obtain a more conservative estimate.

## Conclusion

The reduction of health-care costs in the context of OPT suggests that – under naturalistic conditions – OPT as part of the health-care system can be seen as an investment, which will pay off in the future in terms of reduced costs for inpatient treatment and a decrease of the number of work disability days.

## Ethics Statement

Ethics Commission: Jena University Hospital

The Institute of Psychosocial Medicine and Psychotherapy received anonymous dataset. Planning of the study, sampling, recruitment, and data assessment have not been performed by our institute, but rather by our ordering customer which provided the anonymous dataset.

## Author Contributions

UA and BS designed the research question for these analyses. EB, IP, FH, EA, AF, and DK conceived, designed, and implemented the QS-Psy-Bay study. UA and RS prepared the data for analyses. UA analyzed the data; discussion of results was performed by UA, BS, AZ, EB, IP, AF, DK, and RS. UA drafted the manuscript under supervision of BS. All authors read the manuscript and gave critical comments. All authors approved the final version of the manuscript.

## Conflict of Interest Statement

The authors declare that the research was conducted in the absence of any commercial or financial relationships that could be construed as a potential conflict of interest.

## Supplementary Material

**Multiple studies examined health-care costs before and after outpatient psychotherapy in Germany. The sample size varied between *N* = 47 and *N* = 666 (see Table S1 in Supplementary Material). Long-term psychoanalytic psychotherapy was most often examined. All studies reported cost reductions. But, the amount of reduction varied from study to study (e.g., reduction of work disability days from 3.0 to 19.6 days/year)**.
