# Supplementary material for: Outpatient Psychotherapy Reduces Health-Care Costs: A Study of 22,294 Insurants over 5 Years
Source: Front Psychiatry. 2016 Jun 13;7:98. doi: 10.3389/fpsyt.2016.00098 (PMC4904013; doi:10.3389/fpsyt.2016.00098)
Supplement: Supplementary file 1 — Multiple studies examined health-care costs before and after outpatient psychotherapy in Germany. The sample size varied between N = 47 and N = 666 (see Table S1 in Supplementary Material). Long-term psychoanalytic psychotherapy was most often examined. All studies reported cost reductions. But, the amount of reduction varied from study to study (e.g., reduction of work disability days from 3.0 to 19.6 days/year). [file table_1.pdf]

Table A.1. Studies related to cost reduction in the context of outpatient psychotherapy (only studies from the German health care system).

Study	Treatment description	Sample	Effect
Thomas & Schmitz (1993)	Unspecified mixture of CBT, PDP, PP	*N* = 240	1 st year before vs. 1 st year after OPT:<br>• Reduction of 5.7 WDD
Breyer et al. (1997)	100% PP	*N* = 604	1 st year before vs. 1 st year after OPT:<br>• Reduction of 8.6 WDD
Dossmann et al. (1997)	100% PP	*N* = 666	Begin of OPT vs. end of therapy (annual totals based on patient reports):<br>• Reduction of 6.0 WDD<br>• Reduction of 2.2 DH
Keller et al. (2001)	100% long-time PP (no. sessions >100); 46% depression, 14% phobic or anxiety disorder, 17% personality disorder, 23% other	*N* = 47	1 st year before vs. 1 st year after OPT:<br>• Reduction of 6.4 WDD<br>• Reduction of 6 DH
Jacobi (2001), subsection 4.4 [(a)]	100% CBT for anxiety disorder	*N* = 67	2nd years before vs. 1 st year after OPT:<br>• 99.0% reduction of WDD from 19.8 to 0.2 days<br>• 25.4% reduction of DH from 21.6 to 1.8 days<br>• 92.1% reduction of contacts to general practitioner
Beutel et al. (2004)	100% PP	*N* = 255	1st year before vs. 1st year after OPT (based on patient reports):<br>• Reduction of WDD from 8.2 to 5.2<br>• Reduction of medical consultations from 5.8 to 4.5<br>• Reduction of DH from 1.8 to 0.6
Kraft et al. (2006) [(b)]	49% CBT (sessions *M* = 28.9), 51% PDP (sessions *M* = 38.3); 48% depression, 16% phobic or anxiety disorder, 5% personality disorder, 31% other	*N* = 176	1st year before vs. 1st year after OPT:<br>• 6.7% reduction of medical costs from €3717.92 to €3468.47<br>• 37.1% reduction of DH from 4.9 to 3.1 days<br>1st year before vs. 2nd year after OPT:<br>• 15.9% reduction of medical costs from

	€3717.92 to €3128.55<br>• 47.1% reduction of DH from 4.9 to 2.6 days<br>2nd years before vs. 2nd years after OPT:<br>• 12.7% growth of medical costs from €2775.61 to €3128.55<br>• 13.0% reduction of DH from 3.0 to 2.6 days

Note: [a] pre-treatment costs refer to two years, no data for 1 year before reported; [b] no annual totals reported, we estimated annual totals based on mean half year sums reported in Kraft et al. (2006, Figure 2, p. 244, and Table II, p. 245); abbreviations: OPT = outpatient psychotherapy, CBT = cognitive behavior psychotherapy, PDP = Psychodynamic Psychotherapy, PP = psychoanalytic psychotherapy, WDD = work disability days, DH = hospitalization days.
